# A mass spectrometry-based assay using metabolic labeling to rapidly monitor chromatin accessibility of modified histone proteins

**DOI:** 10.1038/s41598-019-49894-4

**Published:** 2019-09-20

**Authors:** Simone Sidoli, Mariana Lopes, Peder J. Lund, Naomi Goldman, Maria Fasolino, Mariel Coradin, Katarzyna Kulej, Natarajan V. Bhanu, Golnaz Vahedi, Benjamin A. Garcia

**Affiliations:** 1 Epigenetics Institute, Department of Biochemistry and Biophysics, Perelman School of Medicine, University of Pennsylvania, Philadelphia, PA 19104 USA; 2 Laboratório Especial de Ciclo Celular, Center of Toxins, Immune Response and Cell Signaling - CeTICS, Instituto Butantan, São Paulo, 05503-900 Brazil; 3 Epigenetics Institute, Institute for Immunology, Department of Genetics, Perelman School of Medicine, University of Pennsylvania, Philadelphia, PA 19104 USA; 4 Biochemistry and Molecular Biophysics Graduate Group, Perelman School of Medicine, University of Pennsylvania, Philadelphia, PA 19104 USA; 5 Immunology Graduate Group, Perelman School of Medicine, University of Pennsylvania, Philadelphia, PA 19104 USA; 6 Present Address: Department of Biochemistry, Albert Einstein College of Medicine, Bronx, NY 10461 USA

**Keywords:** Histone post-translational modifications, Proteomics, Post-translational modifications, Epigenetics

## Abstract

Histone post-translational modifications (PTMs) contribute to chromatin accessibility due to their chemical properties and their ability to recruit enzymes responsible for DNA readout and chromatin remodeling. To date, more than 400 different histone PTMs and thousands of combinations of PTMs have been identified, the vast majority with still unknown biological function. Identification and quantification of histone PTMs has become routine in mass spectrometry (MS) but, since raising antibodies for each PTM in a study can be prohibitive, lots of potential is lost from MS datasets when uncharacterized PTMs are found to be significantly regulated. We developed an assay that uses metabolic labeling and MS to associate chromatin accessibility with histone PTMs and their combinations. The labeling is achieved by spiking in the cell media a 5x concentration of stable isotope labeled arginine and allow cells to grow for at least one cell cycle. We quantified the labeling incorporation of about 200 histone peptides with a proteomics workflow, and we confirmed that peptides carrying PTMs with extensively characterized roles in active transcription or gene silencing were in highly or poorly labeled forms, respectively. Data were further validated using next-generation sequencing to assess the transcription rate of chromatin regions modified with five selected PTMs. Furthermore, we quantified the labeling rate of peptides carrying co-existing PTMs, proving that this method is suitable for combinatorial PTMs. We focus on the abundant bivalent mark H3K27me3K36me2, showing that H3K27me3 dominantly represses histone swapping rate even in the presence of the more permissive PTM H3K36me2. Together, we envision this method will help to generate hypotheses regarding histone PTM functions and, potentially, elucidate the role of combinatorial histone codes.

## Introduction

Epigenetics is the study of heritable changes in gene regulation that are not coded in the DNA sequence, a phenomenon playing a fundamental role in a wide range of biological processes including cellular differentiation and disease pathogenesis^1^. In eukaryotic cells, DNA exists in close association with histone proteins to form chromatin. The basic repeating unit of chromatin is the nucleosome, which consists of ~147 bp of DNA wrapped around a histone octamer containing two copies each of the core histones H2A, H2B, H3, and H4. One mechanism of epigenetic regulation involves post-translational modifications (PTMs) added to histones, especially the N-terminal tails. Acetylation, for instance, neutralizes positive charge of lysine, which reduces the electrostatic interaction between the positively charged histones and the negatively charged DNA, thereby decreasing chromatin compaction. Open and closed chromatin have become almost synonyms of actively transcribed and silenced chromatin, respectively^2^. In addition to controlling chromatin compaction^3^, histone PTMs may also regulate genetic processes by serving as binding sites for “reader” proteins, such as transcriptional activators or chromatin remodelers.

Mass spectrometry (MS) is currently the only technique that can identify and quantify in a large-scale manner the relative abundance of histone PTMs^4^. Other techniques, such as Western blotting, ELISA or immunohistochemistry, may be used for histone PTM quantification, but they all rely on specific antibodies, which may not be readily available for some PTMs or may provide inaccurate quantitation due to cross-reactivity or epitope masking^5,6^. Correlating histone PTMs with the local chromatin compaction state is currently only possible with the use of antibody-based techniques. Although chromatin accessibility assays, such as ATAC-seq^7^ and DNase-seq (reviewed in^8^), are not dependent on antibodies, mapping histone PTMs across the genome by ChIP-seq to determine their relationship with accessible chromatin is dependent on the availability of an antibody of sufficient quality. To identify histone PTMs that associate with accessible chromatin in a high-throughput and unbiased manner, we recently developed “gradient-seq”, a method in which chromatin is fractionated over a sucrose gradient based on its resistance to sonication^9^. Using this approach, we successfully isolated large domains of structural heterochromatin and analyzed associated histone PTMs by LC-MS. However, this method is unable to define the histone PTMs associated with more subtle differences in chromatin compaction since it is largely limited to resolving heterochromatin from euchromatin.

The absence of a large-scale method that matches chromatin accessibility with histone PTMs has become more apparent in recent years. More than 400 histone PTMs have been discovered to date^10^, mostly thanks to advances in MS sensitivity. However, functional validation remains a complex task since histone PTMs typically exist in combinations and the same histone PTM may have different genetic outcomes depending on the presence of other histone PTMs^11^. This represents a challenge for antibody-based techniques like ChIP-seq, which normally target single histone marks. Further motivation for a method that relates chromatin accessibility to histone PTMs comes from growing interest in targeting epigenetic pathways for the treatment of cancer^12^. At the end of 2016, 33 epigenetic drugs were FDA-approved for cancer treatment, several of which target histone “writers” or “erasers,” the enzymes that catalyze deposition and removal of histone PTMs. Considering that more than 50% of human cancers have mutations in genes involved in chromatin regulation, and cancer cells routinely use epigenetic processes to evade the immune system and chemotherapy^12^, new quantitative analyses are of great importance to identify unexplored differences between normal versus aberrant chromatin.

Canonical histones are deposited on chromatin during S-phase of DNA replication^13–15^. However, histone synthesis and deposition is a highly dynamic process that also occurs outside of S-phase as a necessity for histone eviction and degradation. Histone eviction is a well-characterized event that primarily occurs in regions of transcriptionally active chromatin, thereby allowing transcription factors and RNA polymerase to access and transcribe the underlying DNA. Nucleosome turnover has been described in multiple publications^16–18^, firmly establishing that nucleosomes are exchanged from chromatin multiple times within a cell cycle. This also indicates that histone modifications are not always removed by erasers and may be removed through nucleosome turnover instead. This was recently demonstrated for the histone mark H3K79me^19^, a PTM catalyzed by DOT1L but without a known demethylase. Another example is replacement of histone H2A with the variant H2A.Z by the SRCAP complex^20^. Rates of histone turnover were estimated by using metabolic labeling coupled to MS in a previous publication by Zee *et al*.^21^ and Scharf *et al*.^22^. In these publications, the turnover rate was estimated for about 50 modified histone peptides, identifying clear differences between peptides belonging to the same histone isoform but with different PTMs. By using stable isotope labeling with amino acids in cell culture (SILAC)^23^ combined with MS, we can quantify the rate of translation of proteins as a complementary quantitative layer to protein abundance^24^. In the case of histones isolated from nuclei, the ratio between light and heavy histones monitors also histone transport to the nucleus, histone incorporation, histone eviction and histone degradation. Interestingly, when monitoring the same protein sequence with different modifications, it is possible to estimate differences in the turnover rates of the differentially modified forms of the protein.

In this work, we analyzed the different turnover rates of histones using 219 different peptides considered different modifications and/or histone variants. As model system, we used the murine T lymphoblast cell line EL4. We found that incorporation of the labeled amino acid into histone peptides bearing specific PTMs correlated with the known association of the histone PTM with accessible chromatin. We thus performed a systematic analysis and validation of the method, which resulted in an accurate approach to define the compaction state of differently modified histones. Specifically, by utilizing stable isotope labeled arginine to measure the rate between “new” and “old” histones. In other words, by labeling the sequence of the histone and not the PTM itself we can monitor the labeling rate of histone proteins that carry a given modification rather than the labeling rate of modifications themselves. This method provides a currently unexplored dimension in the quantitative analysis of histones by proteomics.

## Experimental Procedures

### EL4 cell culture and labeling

EL4 cells (mus musculus lymphoma) were from ATCC and labeled as TIB-39 (batch number: 63681083). EL4 maximum duplication rate is estimated to be 13 hours. EL4 cells were maintained in RPMI 1640 containing GlutaMAX (Gibco 72400–047) and supplemented with 10% serum and 1X Penicillin/Streptomycin (Gibco 15140–122) in a humidified incubator at 37 °C and 5% CO_2_. For labeling experiments, 1 million cells were plated in 1 ml medium in a 24-well plate. Unlabeled (R0) or isotope-labeled (R10, ^13^C_6_^15^N_4_) arginine (Silantes) was then added to 5.75 mM and cells were cultured for 1 to 3 days in the presence or absence of inhibitors. The EZH2 inhibitor UNC1999 and the HDAC inhibitor panobinostat were used at 1 µM and 1 nM, respectively. At the appropriate time points, cells were pelleted, washed once in PBS, and then flash frozen in liquid nitrogen. For the cell cycle synchronization experiment, cells were serum-starved overnight prior to labeling. Unless otherwise specified, all experiments were performed in three biological replicates, where by biological replicate is intended a separate cell culture (using the same original batch).

### Histone extraction, derivatization and digestion

Nuclei were isolated from 30 µl of cell pellet by using Nuclei Isolation Buffer (NIB) as previously described^4^. Two rounds of NIB incubation were performed at a volume buffer:cell pellet of 9:1; the first round 0.2% NP-40 was added to lyse the cell membrane, and the second without NP-40 to remove the detergent from the nuclear pellet. Each step included centrifugation at 1,000 × g for 10 min to pellet the intact nuclei. Next, the pellet was incubated in 0.2 M H_2_SO_4_ for 2 hours, and the supernatant was collected after centrifugation for 10 min at 1,000 × g. Finally, histones were precipitated with 33% trichloroacetic acid (TCA) for 1 hour. The histone pellet was then washed with ice-cold acetone to remove residual TCA.

Histone pellets were resuspended in 20 μL of 50 mM NH_4_HCO_3_ (pH 8.0) and split into two aliquots for digestion into ArgC-like peptides (bottom-up) and intact histone N-terminal tails (middle-down). For the bottom-up preparation, we added to the sample 5 µl of acetonitrile followed by 5 µl of propionic anhydride and 14 µl of ammonium hydroxide and incubated for 20 min at room temperature. The reaction was performed twice to ensure complete derivatization of unmodified and monomethylated lysine residues. Samples were then dried, resuspended in 20 μL of 50 mM NH_4_HCO_3_ and digested with trypsin (Promega) (enzyme:sample ratio 1:20, 2 hours, room temperature). The derivatization reaction was then performed again twice to derivatize peptide N-termini.

Middle-down sample preparation was performed by adding to the histones in NH_4_HCO_3_ 1 μg of GluC (Millipore), and leave the digestion overnight at room temperature. For both sample preparations, samples were desalted by using in-house packed stage-tips and dried using a SpeedVac centrifuge.

### NanoLC−MS/MS for bottom-up histone peptide analysis

Samples were resuspended in 0.1% trifluoroacetic acid (TFA) and injected onto a 75 µm ID × 25 cm Reprosil-Pur C_18_-AQ (3 µm; Dr. Maisch GmbH, Germany) nano-column packed in-house using an EASY-nLC nanoHPLC (Thermo Scientific, San Jose, CA, USA). The nanoLC pumped a flow-rate of 300 nL/min with a programmed gradient from 5% to 28% solvent B (A = 0.1% formic acid; B = 80% acetonitrile, 0.1% formic acid) over 45 minutes, followed by a gradient from 28% to 80% solvent B in 5 minutes and 10 min isocratic at 80% B. The instrument was coupled online with a Q-Exactive (Thermo Scientific, Bremen, Germany) or an Orbitrap Fusion (Thermo Scientific, San Jose, CA, USA) mass spectrometer acquiring data in a data-independent acquisition (DIA) mode as previously optimized^25,26^. Briefly, DIA consisted on a full scan MS (*m/z* 300−1100) followed by 16 MS/MS with windows of 50 *m/z* using HCD fragmentation and detected all in high resolution.

### NanoLC-MS for middle-down intact histone tail analysis

Intact histone N-terminal tails (aa 1–50) were analyzed as previously described^27,28^. Briefly, histone tails were resuspended in 75% acetonitrile (pH 6.0) and loaded onto a 15 cm analytical column (75 µm ID) packed with Polycat A resin (PolyLC, Columbia, MD, 2 µm particles, 1000 Å). Both the nanoHPLC and the mass spectrometer used were the same as for the bottom-up analysis. The nanoLC pumped a flow-rate of 250 nL/min with a programmed gradient of 5 min isocratic at 0% B, followed by 55 to 85% B in 150 min (A = 75% acetonitrile, 20 mM propionic acid, adjusted to pH 6.0 using ammonium hydroxide; B = 15% acetonitrile adjusted to pH 2.5 with formic acid). Spectra were acquired in the Orbitrap Fusion as previously described^29^. Briefly, both MS and MS/MS were performed in the Orbitrap to achieve high resolution. The MS scan was acquired in the *m/z* range 665−710. MS/MS was performed using ETD fragmentation. Nevertheless, only the precursor ion intensity was used for the analysis.

### MS data analysis

All raw files are freely available on https://chorusproject.org at the project no. 1555. DIA data obtained from the bottom-up analysis were searched using EpiProfile 2.0^30^. The peptide relative ratio was calculated using the total area under the extracted ion chromatograms of all peptides with the same amino acid sequence (including all of its modified forms) as 100%. For isobaric peptides, the relative ratio of two isobaric forms was estimated by averaging the ratio for each fragment ion with different mass between the two species. To obtain the % of heavy labeling, we used the intensity of the heavy labeled form divided by the intensity of light + heavy form. Data obtained from the middle-down runs were analyzed manually. We calculated the *m/z* values corresponding to the unlabeled and labeled histone H3.1 peptide (amino acid residues 1–50) at a charge state 8^+^, being this charge state within the acquisition window. We considered the histone peptide as unmodified and with up to 15 methyl equivalents (14.01 Da each). Next, we performed extracted ion chromatography of those *m/z* ions with a mass tolerance of 10 ppm and calculated the % of heavy labeling as performed in the bottom-up analysis. Statistical significance was assessed using a two-tails heteroscedastic t-test (p-value representation * =  < 0.05, ** =  < 0.005, *** =  < 0.0005). When mentioned correlation, Pearson Product-Moment correlation is intended.

### ATAC-seq assay

ATAC-seq was performed as previously described with minor modifications^31^. 50,000 cells were pelleted at 550 × g and washed with 1 ml of PBS, followed by treatment with 50 ml lysis buffer prepared as follows: 10 mM Tris-HCl (pH 7.4, 10 mM NaCl, 3 mM MgCl_2_, 0.1% IGEPAL CA-630. After pelleting nuclei, the pellets were resuspended in 50 ml transposition reaction with 2.5 ml Tn5 transposase (FC-121–1030; Illumina) to tag and fragment accessible chromatin. The reaction was incubated in a 37 °C water bath for 45 minutes. Tagmented DNA was purified using a MinElute Reaction Cleanup Kit (QIAGEN) and amplified with 12 cycles of PCR. Libraries were purified using a QIAQuick PCR Purification Kit (QIAGEN). Libraries were paired-end sequenced (38 bp + 38 bp) on a NextSeq 550 (Illumina). 2 technical replicates were constructed. During the ATAC-seq protocol, we did not size-selected DNA fragments to maximize the library complexity; the fragment size was vastly enriched around 100–200 bp (Fig. S3A).

### ChIP-seq assay

Chromatin samples prepared from 10 million fixed cells were immunoprecipitated with antibodies recognizing H3K27ac (ab4729; Abcam), H3K4me3 (04–745; EMD Millipore), H3K27me3 (07–449; EMD Millipore), H3K36me2 (07–274; EMD Millipore), and H3K9ac (ab4441; Abcam). Antibody-chromatin complexes were captured with agarose protein G–conjugated beads, washed, and eluted. After reversal of cross-linking, RNase and proteinase K treatment were performed and DNA was purified and quantified for library preparation. Input sample was prepared by the same approach without immunoprecipitation. Libraries were then prepared using the UltraTM II DNA Library Prep Kit (NEB). Two replicates were performed for each condition. Indexed libraries were validated for quality and size distribution using a TapeStation 2200 (Agilent). Paired end sequencing (38 bp + 38 bp) was performed on a NextSeq 550.

### RNA-seq assay

RNA was extracted from 300,000 cells using RNAeasy Kit (Qiagen- 74014) according with manufacturer’s instructions and including DNAse digestion. Library were constructed using NEBNext Poly(A) mRNA Magnetic Isolation module and NEB Ultra Directional RNA Library Prep Kit for Illumina (NEB). Libraries qualities were assed with Agilent BioAnalyzer 2100 (Agilent) and quantified using KAPA Library Quantification kit (KAPA Biosystems). Single end sequencing (75 bp) was performed on a NextSeq 500. 2 technical replicates were constructed.

### High-throughput sequencing data processing

ChIP-seq, ATAC-seq and RNA-seq data are available in GEO at the identifier no. GSE125384. STAR v2.5 was used to align ATAC and ChIP-seq. ATAC-seq and ChIP-seq samples were analyzed with the parameters–alignIntronMax 1–alignMatesGapMax 2000–alignSJDBoverhangMin 9999–alignSJoverhangMin 9999–outFilterMultimapNmax 1–outFilterScoreMinOverLread 0–outFilterMatchNminOverLread 0–outFilterMatchNmin 20. Reads aligned to the mitochondrial genome, chromosome x and y, as well as reads mapping to multiple genomic loci were discarded from downstream analyses. Additionally, Picard minimized the PCR amplification bias in ATAC-seq and ChIP-seq. Peak calling for ChIP-seq binding sites for narrow histone modifications were identified by applying macs2 with parameters ‘-p 1e-5–nolambda–nomodel–keep-dup all’ using the corresponding input sample as control resulting in 28441, 20766, and 18574 peaks for H3K27ac, H3K9ac, and H3K4me3. Epic v0.2.9 was used for broad peak calling of H3K27me3 and H3K36me2 ChIP-seq samples with the parameter –false-discovery-rate-cutoff 1e-7 using the corresponding input sample as control resulting in 43,819 and 40,385 peaks respectively. ATAC-seq peaks were identified by applying macs2 with parameters ‘-p 1e-5–nolambda–nomodel–keep-dup all’ resulting in 20,771 peaks. Peak numbers are the result of merging peaks from two technical replicates. Gene models were downloaded from Gencode M11 and the TSS of each gene were extended by 3 kb upstream and 2 kb downstream to overlap with ChIP-seq binding sites to designate promoter proximal and distal peaks. Aligned ATAC-seq reads were counted at ChIP-seq merged peaks for each histone modification with bedtools coverage. Reads/peak were normalized for peak length and reads/100 bp is displayed.

## Results

**Figure 1:**
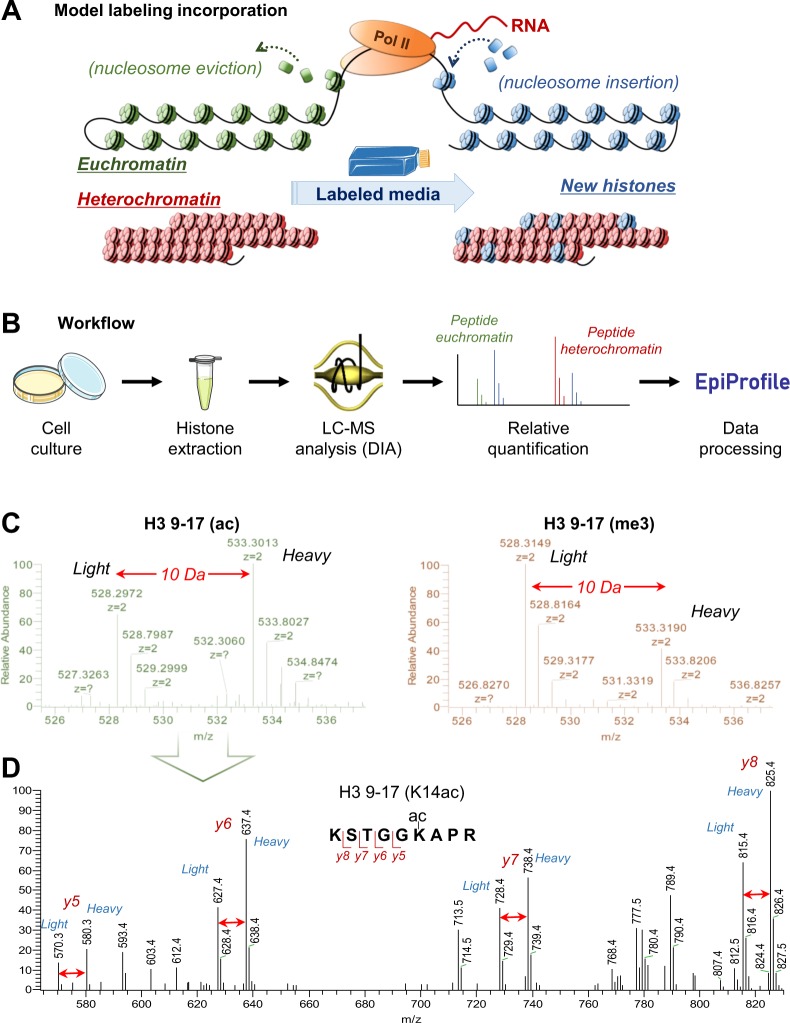
Overview of the workflow and representative result spectra. (**A**) Rationale of the metabolic labeling principle; accessible chromatin has higher nucleosome turnover and thus it is labeled with higher rate as compared to silenced heterochromatin. Labeling is represented with the blue color. (**B**) Schematic of the workflow. Cells are grown in culture for a relatively short amount of time (1–3 days) with selected isotopically heavy amino acids (in this work, arginine) and then processed by using our in-house workflow for canonical histone PTM analysis. Relative quantification (relative PTM abundance and heavy/light ratio) is performed automatically by our in-house software EpiProfile^30^. (**C**) Full MS spectrum of the peptide of histone H3 KSTGGKAPR (aa 9–17) carrying one acetyl group (on the left) or one trimethyl group (on the right). Evidently, the peptide in its trimethylated form has a lower % heavy arginine incorporation. (**D**) MS/MS spectrum of the same peptide carrying one acetyl group, co-isolating the light and the heavy form. The fragment ions can be used to confirm the relative amount of labeling even in presence of isobaric forms (K9ac vs K14ac). Panel A and B were produced, in part, by using Servier Medical Art (http://smart.servier.com/). Servier Medical Art is licensed under a Creative Commons Attribution 3.0 License (CC BY 3.0 license: https://creativecommons.org/licenses/by/3.0/). The color of the images downloaded was modified to fit our figure.

We present a new workflow and data processing method to integrate in a single analysis the relative quantification of histone PTMs and the turnover rate of nuclear histone proteins carrying the given PTM. Canonical histones are synthesized during the S-phase of the cell cycle^32^, but they are then incorporated into the chromatin and modified differently depending on the properties and biological function of the chromatin region. Because of this, it is possible to monitor which chromatin regions have higher rate of activity, due to e.g. transcription, by monitoring the relative percentage of isotopically labeled histone proteins (Fig. 1A). Our approach can quantify the swapping rate on chromatin of histones with different PTMs by quantifying the percentage of isotopically labeled histone peptides. Specifically, our assay labels the sequence of the histone proteins rather than the modifications, being this way independent from the writer/eraser accessibility dynamics. The labeling can be performed on any cell line that can grow for 1–2 days in culture. We verified that cell media do not require special filtration or custom preparation; it is sufficient to spike into the media a 5x concentration of isotopically labeled arginine (compared to normal arginine), so no depletion of the canonical light arginine is required. Calculations to ensure an approx. 5x labeled/unlabeled arginine ratio for the cells were performed as follows: we plated 1,000,000 cells in 1 mL medium. Heavy labeled arginine was 5.75 mM in the medium (~1 mg/mL), while RPMI 1640 medium (Thermo Fisher Scientific) contains about 0.2 mg/mL of unlabeled arginine. We considered the following: (i) one cell contains about 300 pg of proteins, therefore 1,000,000 cells contain about 300 µg of proteins; (ii) arginine occurs in cellular proteins at 5.78% compared to total amino acids^33^; (iii) 3 days cell growth leads to a maximum of 4,000,000 cells at the end of the culture. Together, this corresponds to ~70 µg of unlabeled arginine within the cell proteome. This adds up to 270 µg of unlabeled arginine vs 1 mg of labeled one. This calculation did not consider the proteins present in serum (mix at 10% compared to medium), because serum proteins are not freely available for cellular metabolism. Figure 1B presents the workflow for the analysis of histone peptides; histones are extracted from cells in culture, analyzed by liquid chromatography coupled online to tandem mass spectrometry (LC-MS/MS) and data are processed using our in-house software EpiProfile 2.0^30^.

### Accurate quantification of histone peptide labeling

We manually verified that the labeling incorporation occurs, and it is clearly visible at 2 days of cell growth in heavy arginine media (Fig. 1C). We selected as an example two signals with nearly isobaric mass, i.e. a peptide modified with an acetylated lysine residue (42.010 Da mass addition) or a trimethylated lysine residue (42.047 Da mass addition). The peptide considered, KSTGGKAPR, is from the histone H3 sequence and it spans through the residues 9–17. If acetylated, the modification can occur on the K9 or the K14 residue; the trimethyl group is exclusive for K9. The figure shows that the heavy form of the peptide (with a light-heavy delta of 10 Da, or 5 *m/z*) is more abundant in the acetyl form. This indicates that the histone carrying the acetylated form has a faster accumulation of heavy labeling than the trimethyl one, which is what we would expect from histones associated to more active chromatin. It is important to note that this is apparently in contraposition with the idea that silencing marks like H3K9me3 and H3K27me3 are those being epigenetically inherited during cell duplication^34^. To accurately map the position of the PTM, we used a data-independent acquisition (DIA) method in mass spectrometry, where all the peptides are fragmented at every duty cycle (approximatively every 2 seconds). This enables us to accurately quantify peptides at the fragment ion level, discriminating where the PTM is localized in the labeled form (Fig. 1D). The extraction of 219 (un)modified histone peptides is automatically performed by our in-house software EpiProfile 2.0^30^ (Table S1).

**Figure 2:**
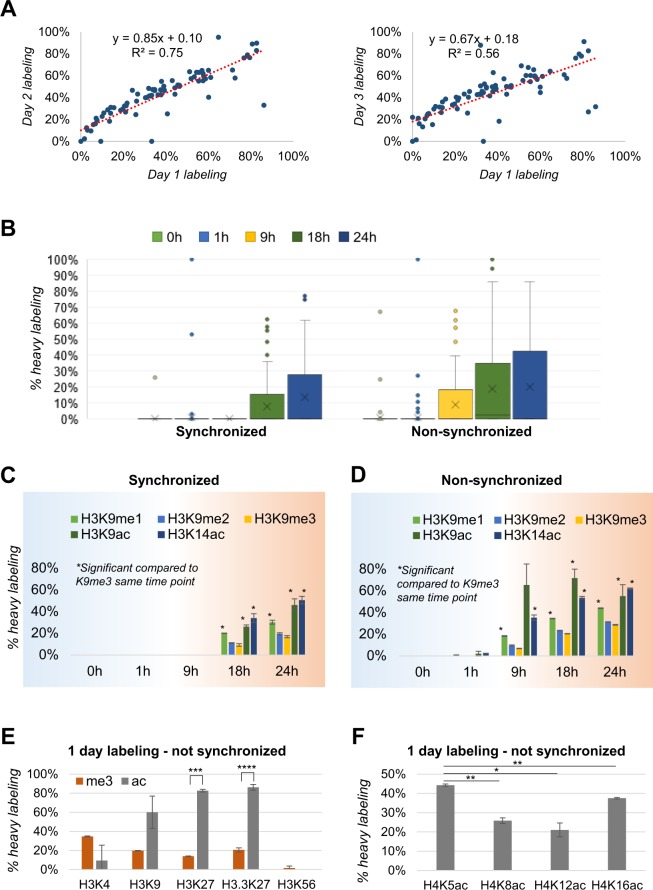
Labeling incorporation as function of time. (**A**) Percentage of labeling incorporation on all detectable 219 peptides after 1 day vs 2 days of growth in labeling media (left) or after 1 day vs 3 days of labeling (right) (correlation p-value 10e-26 and 10e-12, respectively). (**B**) Labeling incorporation on all detectable peptides represented as box plot. (**C**) Labeling incorporation of the peptide of histone H3 KSTGGKAPR (aa 9–17) in all its modified forms. Cells were labeled after being synchronized and (**D**) not synchronized. (**E**) Labeling incorporation of the histone H3 residues hosting the most abundant trimethyl and acetyl marks. The comparison considers only peptides modified with a single PTM; e.g. does not include hybrid modified peptides such as H3K9me3K14ac. (**F**) Labeling incorporation of the histone H4 peptide GKGGKGLGKGGAKR (aa 4–17) modified with a single acetylated residue. Significance represented between H4K5ac and the other marks. All significance estimations are based on a two-tail heteroscedastic t-test (significant when p-value < 0.05).

The labeling incorporation of histone proteins of EL4 cells is proportional to their growth time, as expected. We verified that peptides with the faster turnover at day 1 correlate with those at day 2 and day 3 (Fig. 2A, Table S1), indicating that there is no unexpected change in labeling incorporation rate of the different histones during the cell growth. A fraction of the peptides such as H2AK7ac and H3.3K27ac showed poor correlation across days; these peptides were of low abundance and thus more challenging to accurately quantify. To observe whether labeling incorporation is prevented when cells do not undergo S phase, we arrested cells in G0 by starving them for 18 hours, then released them and collect time points up to 24 hours (Fig. 2B). It is evident that cells undergoing cycle arrest do not produce any significant labeling incorporation until the time point at 18 hours, while those not arrested show readily detectable incorporation already at 9 hours. While theoretically a 50% average labeling would be expected after 1 cell cycle (due to chromatin duplication), it is important to note that the utilized media contains also unlabeled arginine and cells have internal recycling of amino acids.

Next, we focus on the labeling rate of the same peptide sequence carrying different modified forms. We selected as example the peptide of histone H3 KSTGGKAPR (aa 9–17) in all its forms with a single modification (Fig. 2C,D). The form modified as H3K9me3 has a labeling rate significantly smaller than the acetylated forms H3K9ac or H3K14ac, confirmed in both synchronized and unsynchronized cells. This was not surprising and an important first indication that our model (Fig. 1A) is reliable; in fact, H3K9me3 is a well-known marker of structural heterochromatin, where nucleosome turnover is rare^35^. Furthermore, we can observe differential labeling for the different methylation states of H3K9, where the labeling rate observed was K9me1 > K9me2 > K9me3 (Fig. 2C,D). All the residues of histone H3 which can be modified with either me3 or ac showed a much higher labeling incorporation when the acetyl group was present (Fig. 2E). One exception was the residue H3K4me3, as it is well known to be a mark of active promoters^36^. H3K56ac labeling rate could not be properly quantified because of its very low abundance overall. We also investigated the turnover of histone H4, identifying significant differences whether it was acetylated at the residues K5, K8, K12 or K16 (Fig. 2F). While the outcome of histone H3 PTM analysis was expected, not much is known about the differences in histone swapping rate when histone H4 is acetylated on different residues, paving the way to interesting new observations about the differential roles of uncharacterized histone marks.

### Independence between chromatin turnover and cell treatment

**Figure 3:**
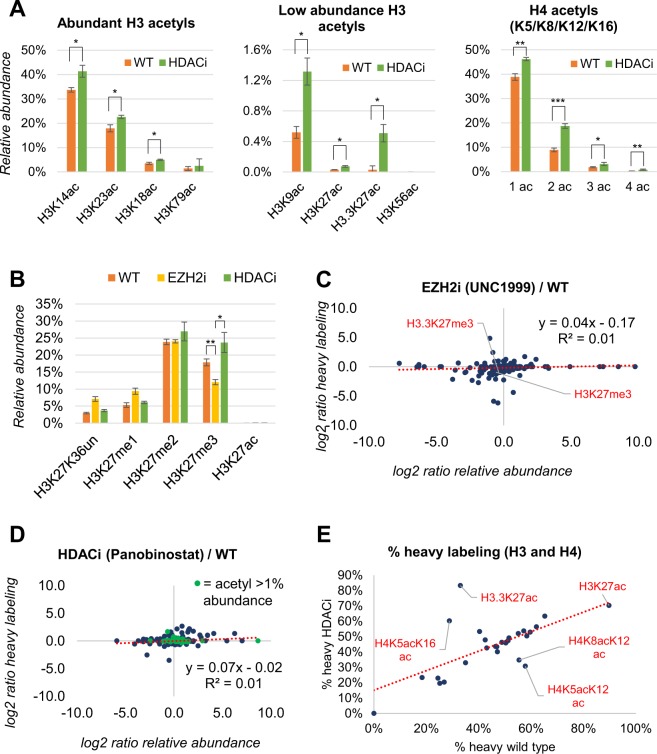
Regulation of PTM relative abundance and labeling rate upon drug treatment. (**A**) Relative abundance of the most abundant acetylations of histone H3 and H4 in wild type and HDAC inhibitor (HDACi) treated cells. (**B**) Relative abundance of representative modified forms of histone H3 regulated during treatment with inhibitors. All data were extracted after 2 days of labeling. All significance estimations are based on a two-tail heteroscedastic t-test (significant when p-value < 0.05). (**C**) Correlation analysis of fold change regulation (not significant) of the relative abundance of histone peptides after treating cells with EZH2 inhibitor (x-axis) vs the fold change of labeling incorporation (y-axis). The y-axis shows very minor changes, indicating that the regulation of PTM abundance is independent from the labeling incorporation. (**D**) Same correlation analysis using the HDAC inhibitor Panobinostat (not significant). (**E**) Labeling incorporation of the major acetylated peptides of histone H3 and H4 in wild type and HDACi treated cells.

Protein production, and by consequence protein turnover, changes upon cell stimulation. Protein turnover is frequently used as complementary quantitative dimension to protein abundance to assess differences of phenotypes between two conditions^24^. Our hypothesis is that the differences in turnover we observe in the modified histones is an intrinsic property of the local swapping rate of histones on chromatin, and therefore it is independent from changes of the cell state. To test this assumption, we treated EL4 cells with two drugs that drastically affect the abundance of specific PTMs. UNC1999 inhibits EZH2, which catalyzes H3K27me3; Panobinostat inhibits histone deacetylases (HDACs), responsible for erasing acetylations on histone proteins. First, we verified that the relative abundance of acetylations increased upon HDAC inhibitor treatment; we observed that all the quantified histone H3 and H4 acetylations are overall more abundant in cells treated for two days with the inhibitor (Fig. 3A). Next, we quantified a statistically significant reduction of the mark H3K27me3 in presence of the EZH2 inhibitor UNC1999 (Fig. 3B). The reduction of H3K27me3 was counterbalanced by an increase in the relative abundance of the unmodified peptide form and the H3K27me1 form. Notably, we also compared the reproducibility of the measurements in terms of peptide relative abundance and percentage of labeling incorporation (three biological replicates), demonstrating that labeling incorporation is not only a precise measurement, but it is a significantly more precise quantitative measurement than PTM relative abundance (Fig. S1A). From a mathematical perspective, this is also because the labelling incorporation is only taking two values into account (heavy/light) while PTM relative abundance in the simplest case is generally considering four to five values (e.g. unmod, me1/2/3, ac peptide forms) leading to an intrinsically higher error propagation.

Once we assessed that the drugs are effective on our cell line, we analyzed the effect on the turnover rate of histones modified with different PTM states. We did not observe any correlation between the changes in relative abundance of the PTMs and the turnover of the peptides when cells were treated with the EZH2 inhibitor (Fig. 3C). When comparing with the HDAC inhibitor, results appeared similar (Fig. 3D). By using the same scale for the axes of the scatterplots, it is evident that fold changes of PTM abundances have a dynamic range of about 32-fold (2^5) wider than changes in turnover rate. This indicates that the labeling rate of a histone proteoform is a more conserved property than its abundance, suggesting that the chromatin state where a given histone mark reside is independent from how widespread it is on the chromatin. As confirmation that labeling rate is independent from treatment, we observed the PTM H3K27ac occurring on the histone with the highest turnover in wild type and HDACi treated cells (Fig. 3E, top right), despite this treatment clearly affects the abundance of histone acetylation (Fig. 3A). Moreover, we ruled out that labeling rate and abundance are correlated by calculating the correlation value of all replicates for each of the three days of incubation of the untreated sample (Fig. S2); we observed an average correlation value for PTM abundance of 0.94, an average correlation for labeling rate of 0.73, and an average correlation between abundance vs turnover of same sample of 0.13. On the other hand, three different combinations of doubly acetylated histone H4 had different turnover rates when comparing the wild type and the HDAC inhibitor treated cells. This is noteworthy, since the doubly acetylated histone H4 showed an overall higher abundance in HDAC inhibited cells (Fig. 3A, right); however, only the combination H4K5acK16ac had increased turnover on the chromatin during the drug treatment. The full table on the 219 peptides quantified in terms of relative abundance and turnover rate for the three conditions (WT, EZH2i and HDACi) collected after 1–3 days of incubation is available as Table S1 and Fig. S7. Together, our data demonstrate that histone labeling rate is an independent quantitative dimension compared to PTM relative abundance, because we observed no correlation between changes in PTM abundance and peptide labeling rate upon drug treatment.

### Validation of the correlation between labeling and histone swapping on chromatin by genomics analysis

**Figure 4:**
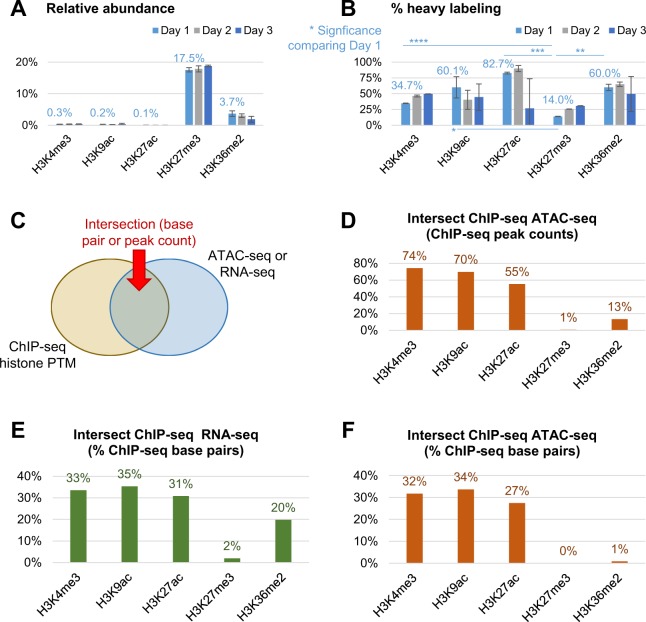
Validation of the link PTM – chromatin state via genomics analysis. (**A**) Relative abundance of the five selected PTMs of histone H3 at day 1 to 3 of EL4 cell culture. (**B**) Labeling incorporation of the five representative marks. P-value is calculated by comparing H3K27me3 with the other PTMs at day 1. All significance estimations are based on a two-tail heteroscedastic t-test (significant when p-value < 0.05). (**C**) Description of how the following data were retrieved; panels D–F include the intersection of the reads obtained both by ChIP-seq and either ATAC-seq or RNA-seq. (**D**) Number of peaks intersecting in ChIP-seq and ATAC-seq data. (**E**) Intersection (counted base pairs) of genomics regions covered by both ChIP-seq and RNA-seq or (**F**) ATAC-seq.

To demonstrate that the PTMs occurring on peptides with the highest turnover rate are indeed marks of active transcription, we performed genome-wide mapping of five selected marks of histone H3 with characterized function in terms of modulating gene expression. We selected three active marks (H3K27ac, H3K4me3 and H3K9ac), one mark well-known for its role in defining constitutive heterochromatin (H3K27me3), and one PTM known to occupy transcribed genes but with a still ambiguous link to gene regulation and DNA repair (H3K36me2)^37^. H3K36me2 has been cited to both activate and inhibit transcriptional elongation^33^; this ambiguity might be due to the limitations of antibody-based techniques to discriminate genome-wide regions occupied by just H3K36me2 rather than its co-existence with silencing marks, e.g. H3K27me3K36me2. First, we selected peptides containing exclusively each of these PTMs, disregarding those with multiple modifications. The peptide modified with H3K27me3 (aa 27–40) was estimated to be of high relative abundance (Fig. 4A), suggesting that this PTM is the most abundant genome-wide. The average % of labeling of these peptides across the three days was as following: K27ac (66.5%), K36me2 (58.2%), H3K9ac (48.4%), H3K4me3 (43.6%) and K27me3 (23.5%) (Fig. 4B). We therefore considered as “active” PTMs those labeled > 40%, which included all analyzed marks but H3K27me3. As expected, low abundance peptide such as H3K27ac had wider error bars due to detection limits; error bars were overall wider at day 3 due to the barely detectable unlabeled form of the peptide.

By using a similar approach as previously described^38^, we intersected regions modified with the above five histone modifications as assessed by chromatin immunoprecipitation followed by high throughput sequencing (ChIP-seq) with genomic regions detected using the assay for transposase-accessible chromatin sequencing (ATAC-seq) (Fig. 4C) and RNA-seq (Fig. 4D). As expected, the ATAC-seq assay maps both nucleosome-free and nucleosomal regions (Fig. S3A)^7^. To estimate which PTMs reside on open chromatin domains, we intersected the regions mapped by ATAC-seq with those mapped by ChIP-seq. By counting the percent of ChIP-seq peaks that had at least a 1 base pair intersection with an ATAC-seq peak, we show that H3K4me3, H3K9ac and H3K27ac occupy accessible chromatin regions (Fig. 4D), in correlation with MS data (Fig. 4B). This was not surprising, also because H3K9ac and H3K27ac showed high correlation in mapped reads by comparing ChIP-seq results (Fig. S3B). H3K36me2 peaks had poor intersection (~13%) with the peaks mapped by ATAC-seq, which did not correlate well with the MS results. This was likely because ATAC-seq reads are highly enriched at distal enhancers and promoter regions, while H3K36me2 is more abundant in gene bodies (Fig. S4). Next, we repeated a similar analysis, and intersected individual RNA-seq reads with ChIP-seq peaks, reporting the resulting % of ChIP-seq total bp that intersected with an RNA-seq read. With this approach we obtained a closer correlation between MS and genomics results (Fig. 4E). For completeness, we repeated the same type of intersection (% bp intersection) between ChIP-seq and ATAC-seq (Fig. 4F), obtaining very similar results as the intersection with peaks (Fig. 4D). This suggests that the labeling rate of histone proteins we observe is more relatable to the active transcription of these domains (requiring nucleosome eviction and replacement^16–18^) rather than chromatin regions that are simply accessible. Violin plots displaying the coverage of ChIP-seq peaks with ATAC-seq or RNA-seq reads (normalized for ChIP-seq peak length) are shown in Fig. S5A,B. Together, we here demonstrated using genomics strategies that the measured labeling incorporation is correlated with the transcription rate of the respective genomics loci. Correlation was not exact for the two datasets, but it is important to reiterate that the labeling rate of the analyzed PTMs refers to peptides specifically modified with only one given modification, while ChIP-seq cannot discriminate histone proteins modified with single vs multiple PTMs.

The ultimate validation that the differential labeling incorporation is due to the transcriptional activity of the polymerase could be assessed by blocking the transcription of the cell. We treated cells with Actinomycin D, a drug responsible for transcriptional inhibition^39^ (Fig. S8). However, since transcription is a critical function of the cell, inhibiting transcription has affected cell viability, as also previously demonstrated^40^. Our results showed that cells treated with actinomycin D have just lower incorporation of heavy arginine, without significant differences across PTMs.

### Chromatin compaction state of histones modified by co-existing PTMs

**Figure 5:**
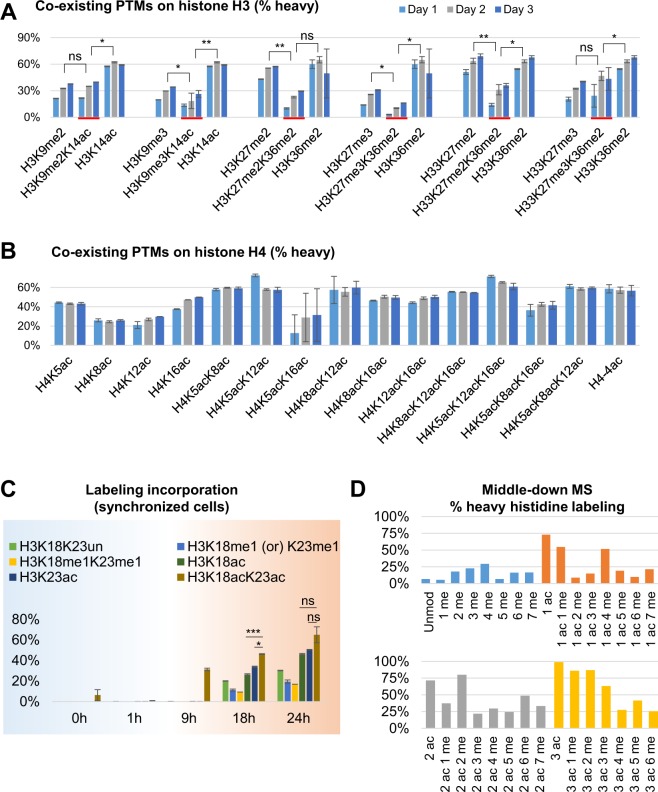
Analysis of labeling incorporation of multiply modified histone proteins. (**A**) Relative labeling incorporation at day 1–3 of the two peptides of histone H3 with the largest number of combinatorial isoforms, KSTGGKAPR (aa 9–17) and KSAPATGGVKKPHR (aa 27–40). Highlighted in red, peptide carrying both flanking modifications. P-value is calculated as paired t-test across the three days. All data were extracted after 2 days of labeling. All significance estimations are based on a two-tail heteroscedastic t-test (significant when p-value < 0.05). (**B**) Same representation for all the modified states of the peptide GKGGKGLGKGGAKR of histone H4, containing all the amino acid residues with the highest amount of acetylation. (**C**) Example of co-existing acetylations on the peptide of histone H3 KQLATKAAR (aa 18–26). The doubly acetylated form has higher labeling incorporation rate. (**D**) Relative incorporation of heavy labeled histidine of the differently modified forms of histone H3 N-terminal tails.

Histones are the most densely modified proteins in eukaryotes^10^. In fact, almost all known protein PTMs have been found on histones as well. Analyses of histone PTMs extracted in bulk from eukaryotic cells have revealed that the relative abundance of fully unmodified histone tails is a tiny minority of the total (e.g.^41,42^). Because of this, histone PTMs are more frequently present in co-existing patterns rather than isolated, in a sort of “histone code”^43^ that is still far from being fully deciphered. With our technique, we analyzed histone peptides carrying co-existing histone PTMs. First, we selected two peptides of the canonical histone H3 sequence KSTGGKAPR (aa 9–17) and KSAPATGGVKKPHR (aa 27–40), and one sequence typical of the histone variant H3.3 KSAPSTGGVKKPHR (aa 27–40) (Fig. 5A). These peptides contain the sites K9/K14 and K27/K36, respectively. Above the detection limit, we identified and quantified six binary modified histones containing a PTM known to be enriched in condensed heterochromatin (H3K9me2/3 or H3K27me2/3) and one typically linked to active euchromatin (H3K14ac or H3K36me2). All experiments revealed that the peptides containing both active and repressive PTMs had significantly lower labeling incorporation than peptides with just the active mark (Fig. 5A), indicating that the repressive mark has a dominant effect on chromatin condensation over the co-existing active marks. Results were confirmed by the narrow variation of the three biological replicates (error bar representing standard deviation), and the reproducibility over three days of harvesting, leading to a total of 9 different cell culture collections. To validate these observations, we used our ChIP-seq data of H3K27me3 and H3K36me2, two histone marks already characterized to have antagonistic effects^44^. We intersected the two datasets and obtained the genomics loci where the two PTMs co-localize (Fig. S5C,D). As previously described^45–47^, only a small number of genomic loci could be identified as being modified by both H3K27me3 and H3K36me2 (around 400 regions), suggesting a limitation in utilizing ChIP-seq to study these co-occurring PTMs. The ATAC-seq intersection with ChIP-seq appears to be higher for the binary mark than the individual ones (Fig. S5C), but results are likely not conclusive because of the very low count of mapped peaks. By measuring RNA-seq read coverage at the few obtained genomics loci of the two PTMs we observed that the transcriptional rate of these regions was in this order: H3K36me2 > H3K27me3K36me2 > H3K27me3 (Fig. S5D). This is not an ideal method to define the transcriptional rate of chromatin loci occupied by multiple PTMs, but the trend demonstrates a remarkable difference between H3K36me2 and H3K27me3K36me2, as pointed out by MS results. Interestingly, in almost all cases (Fig. 5A) it appears that the histones hosting the co-existing marks have a turnover always more similar to those where only the repressive mark is present, suggesting that repressive marks play a dominant role in defining chromatin state when active and repressive PTMs are present. A larger array of ChIP-seq experiments could demonstrate this trend for a broader number of repressive/active PTM pairs.

To rule out that the low turnover is not just simply correlating with the more complex PTM pattern we analyzed the peptide of histone H4 GKGGKGLGKGGAKR (aa 4–17) carrying four modifiable lysine residues (Fig. 5B), the peptide of histone H3 KQLATKAAR (aa 18–26) containing the residues K18/K23 (Fig. 5C) and the intact histone H3 N-terminal tails using middle-down MS (Fig. 5D, described in the next paragraph)^48^. The isobaric combinations of co-existing PTMs on the histone H4 peptide were resolved by using our in-house software EpiProfile 2.0^30^; results showed that the PTMs H4K5ac and H4K16ac were those occupying the protein with the fastest turnover (Fig. 5B). However, other combinations did not present the same labeling incorporation. As expected, the trend indicated that the labeling incorporation was on average higher on peptides with a higher degree of acetyl groups (H4–4ac), although it was not possible for us to assess a statistical difference due to the variability of labeling rate for doubly and triply acetylated peptides. On the other hand, a clear difference was present for the bivalent acetylation H3K18acK23ac (Fig. 5C). This peptide showed a significantly higher labeling rate than the peptide carrying only H3K18ac or H3K23ac in all time points analyzed for EL4 cells synchronized by starvation. This was not surprising, as hyperacetylated chromatin is known to be more related to gene activation, but it does rule out the possibility that lower labeling incorporation is due to the number of PTMs that need to be deposited on a given histone.

A major limitation of our approach is the analysis of relatively short peptide sequences (4–20 amino acids). This is an intrinsic issue of the bottom-up MS approach, the most widely adopted workflow for proteomics in general. To demonstrate that acetylation is a major driver of histone swapping rate in general for histone H3, we cultured EL4 cells for middle-down MS analysis^29^. Middle-down MS is the analysis of intact histone N-terminal tails (50–60 amino acid peptides). For this experiment, we grew EL4 cells in a media where 5x isotopic histidine was spiked into the media, as it is the only essential amino acid present in a single copy on the histone H3 N-terminal tail. Labeling amino acids that are present in multiple copies in a peptide sequence leads to smears of isotopes that make the MS analysis more complex^49^. We previously demonstrated that middle-down MS can be performed with metabolic labeling^29^. Here, due to the newer complexity of histidine labeling, we extracted only the precursor masses (light and heavy labeled) of the polypeptides (Fig. 5D); this did not allow us to define the exact residue localization of the PTMs, but we could define the relative abundance of histone H3 tails with different PTM equivalents. Results demonstrated that hyperacetylated histone H3 tails were the ones with the highest labeling rate, indicating once again that these histones are those more accessible and with the highest turnover on chromatin. The peptides with the lowest labeling rate were those hypermethylated and, interestingly, the fully unmodified form. This indicates that histone H3 proteoforms with a completely unmodified N-terminal tail are unlikely to occupy actively transcribed genomics loci. Together, our new methodology demonstrated to provide a new quantitative layer in histone PTM analysis (histone swapping rate), something not currently achievable with any other method in a large-scale manner.

## Discussion

We presented a simple to apply, novel workflow to quantitatively determine the chromatin state associated with histone PTMs. This work was inspired by the challenge of defining the potential biological role of hundreds of identified histone marks, a list that is continuously increasing due to the higher sensitivity of MS and the use of unbiased software to decipher MS spectra (e.g.^50^). Changes in relative abundance of histone marks between phenotypes are frequently of difficult interpretation, especially when there is no previous knowledge about the biological function of the given PTM. In addition, very little is known about the role on chromatin with combinatorial histone marks, even though there is now agreement that histones are frequently decorated by multiple PTMs^41,42^ and that those PTMs have cross-talk that affect each other’s biological role^51^. MS is the method of choice to identify and quantify all histone PTMs in a large-scale manner, but it does not reveal the genome localization of these marks nor other quantitative dimensions that assist the interpretation of their biological role. In a period where novel histone PTMs (e.g.^52–54^) and histone sequence mutations (e.g. H3K27M^55^) are discovered at an impressive pace, it has become necessary to find a faster way to define the potential activity of these hypermodified histones.

The protocol we presented is of simple applicability, as it only requires that cells are grown for 18–24 hours in canonical media with the addition of isotopically labeled arginine (here 5x of canonical arginine or histidine). The MS workflow we performed is identical to our current standard^25^, which includes derivatization of histones with propionic anhydride, trypsin digestion and LC-MS/MS using DIA acquisition. Together, this implies that histone peptide labeling rate is an “almost free” extra quantitative dimension compared to the canonical analysis of histone peptides via MS. We demonstrated that the labeling rate correlates with the active chromatin state where these PTMs reside; this was proved by using as reference PTMs with characterized biological function and by overlaying the genome-wide mapping of selected histone marks with data defining chromatin accessibility (ATAC-seq) and gene expression (RNA-seq) (Fig. 4B–F). In addition, we confirmed widely accepted knowledge in chromatin biology stating that hyperacetylated histones are enriched on actively transcribed chromatin regions (Fig. 5B–D). Interestingly, the swapping rate of nucleosomes could be monitored also in events of replication and chromatin repair, so this protocol could be tuned to analyze complementary aspects compared to transcription.

We have considered whether the heavy labeling incorporation might have been biased by the different kinetics of histone modification on newly incorporated histones upon replication. Our data seem to dispute this aspect for multiple reasons. First, if PTM kinetics was the main factor defining the labeling rate of the histone peptides, the peptides with the most labeling would be the unmodified ones (no kinetics is faster than no modifications). This is clearly not the case, as exemplified in Fig. 5C,D. In addition, assuming that PTM catalysis is the driver of the observed labeling rate, we would at least expect that the same PTM type has comparable labeling in our data. However, we show that e.g. acetylation and trimethylation can have opposite rates on different residues. For instance, Fig. 2E shows that the residues H3K9 and H3K27 have higher labeling on histones modified with acetylation, while H3K4me3 has higher labeling rate than H3K4ac. Finally, if PTM deposition kinetics was the main factor, then it would be logical to observe a faster turnover of histones with a few modifications as compared to many. However, Fig. 5D shows that histones with five modifications, e.g. 3ac + 2me, have higher labeling rate than histones carrying 1 me or 1 ac.

A potentially interesting application of this methodology would be investigating the differential turnover of histone variants. Unfortunately, comparing the labeling rate across proteins is feasible only if different histone proteins are synthesized with the same rate and amounts. If not, comparing the labeling rate of differentially modified sequences is biased towards the synthesis of the histone itself. Our data indicate that the relative abundance of the histone variants can be estimated (Fig. S9A), and e.g. the canonical histone H3 sequence vs H3.3 has comparable labeling rates (Fig. S9B). Interestingly, we did not observe remarkable differences considering the same modification for the different histone variants (Fig. S9C), suggesting that the accessibility of those histones is mostly depending on how they are modified rather than themselves. Unfortunately, this cannot be ultimately demonstrated until canonical H3 isoforms and H3.3 can be expressed at the same rate.

The comparison we carried out between our MS labeling incorporation protocol and the genomics dataset is limited by the biases and limitations between two techniques. In fact, by selecting e.g. the MS labeling incorporation of the peptide modified only as H3K36me2, we disregarded peptides modified with all methylation states of H3K27 (Figs 4B and S1C,D). However, the antibodies used for ChIP-seq cannot discriminate histones modified only by H3K36me2 or combinations of this PTM with all methyl states of H3K27. Therefore, the labeling incorporation observed e.g. for H3K36me2 cannot be directly compared with the genomics loci mapped by the antibody anti-H3K36me2, as a large fraction of histones modified with H3K36me2 contain also other PTMs such as H3K27me (Fig. S1C,D). It is also clear from the MS analysis that the abundance of multiply modified histones overcomes single modifications (Fig. S1B–D), implying that many of the identified regions by ChIP-seq are likely co-modified. We considered this limitation and used co-modified histone peptides to identify novel potential roles of combinatorial PTMs. By isolating the ChIP-seq peaks where both H3K27me3 and H3K36me2 intersected, we identified the most enriched pathway as “signal transduction” (Fig. S6), which we did not observe when plotting all genes mapped by H3K27me3 and H3K36me2 peaks (data not shown). This suggests that chromatin domains occupied by co-existing marks might include genes where a faster and more dynamic regulation is required compared to those where a single histone PTM is present. To validate this observation, a dedicated project will be required.

More broadly speaking, MS is already being used to investigate chromatin organization. Hydrogen/deuterium exchange (H/D-X)^56^, open-ended cross-linking (XL)^57^ and oxidation of amino acid residues^58^ are methods developed to discriminate the accessible regions of a protein as compared to the hidden core parts. However, none of these are yet suitable for very large macromolecules like the intact chromatin. Therefore, we speculate that applications of this methodology are vast. First, this method can be exploited to investigate the compaction state of chromatin modified by acetylation vs other acyl marks such as butyrylation, propionylation and many other derivatives of the β-oxidation of fatty acid that are now center of attention in the link between epigenetics and metabolism^59^. Novel discoveries about histone mutations (e.g. H3K27M^55^) are also just the beginning of a new field of “oncohistones” where new studies will be necessary to understand the role of these mutations on the chromatin state. In this regard, the transcription rate on domains modified with those mutations could provide essential insights regarding how these mutations affect enzyme binding and overall aberrant chromatin compaction. Finally, the chromatin state driven by combinatorial PTMs vs singly modified histones could pave the way to the prioritization of drug targets; a histone mark that drives a chromatin state could potentially be a more appealing target than a histone mark present in many compaction states depending on which other marks co-exist on the same sequence.

In conclusion, in the era of big data we think that any complementary quantitative dimensions can play a fundamental part in deciphering normal vs disease state. Chromatin state is highly important, as it regulates how genes are recognized and expressed. Associating a quantitative estimation of the swapping rate of modified histones might potentially revolutionize how we interpret MS-based histone results and lead to new ways to generate data-driven hypotheses.

## Supplementary information

Supplementary Figures

Supplementary Table 1
