# Supplementary material for: A mass spectrometry-based assay using metabolic labeling to rapidly monitor chromatin accessibility of modified histone proteins
Source: Sci Rep. 2019 Sep 20;9:13613. doi: 10.1038/s41598-019-49894-4 (PMC6754405; doi:10.1038/s41598-019-49894-4)
Supplement: Supplementary file 1 — Supplementary Figures [file 41598_2019_49894_MOESM1_ESM.pdf]

Supplementary figures

A mass spectrometry-based assay using metabolic labeling to rapidly monitor chromatin accessibility of modified histone proteins

Simone Sidoli[1§], Mariana Lopes[1,2], Peder J. Lund[1], Naomi Goldman[3,5], Maria Fasolino[3], Mariel Coradin[1,4], Natarajan V. Bhanu[1], Katarzyna Kulej[1], Golnaz Vahedi[3] and Benjamin A. Garcia[1*]

[1]*Epigenetics Institute, Department of Biochemistry and Biophysics, Perelman School of Medicine, University of Pennsylvania, Philadelphia, PA, 19104, USA*
[2]*Laboratório Especial de Ciclo Celular, Center of Toxins, Immune Response and Cell Signaling - CeTICS, Instituto Butantan, São Paulo 05503-900, Brazil*
[3]*Epigenetics Institute, Institute for Immunology, Department of Genetics, Perelman School of Medicine, University of Pennsylvania, Philadelphia, PA 19104, USA*
[4]*Biochemistry and Molecular Biophysics Graduate Group, Perelman School of Medicine, University of Pennsylvania, Philadelphia, PA, 19104, USA*
[5]*Immunology Graduate Group, Perelman School of Medicine, University of Pennsylvania, Philadelphia, PA, 19104, USA*
[§]Current affiliation: Department of Biochemistry, *Albert Einstein College of Medicine, Bronx, NY 10461, USA*

Figure S1

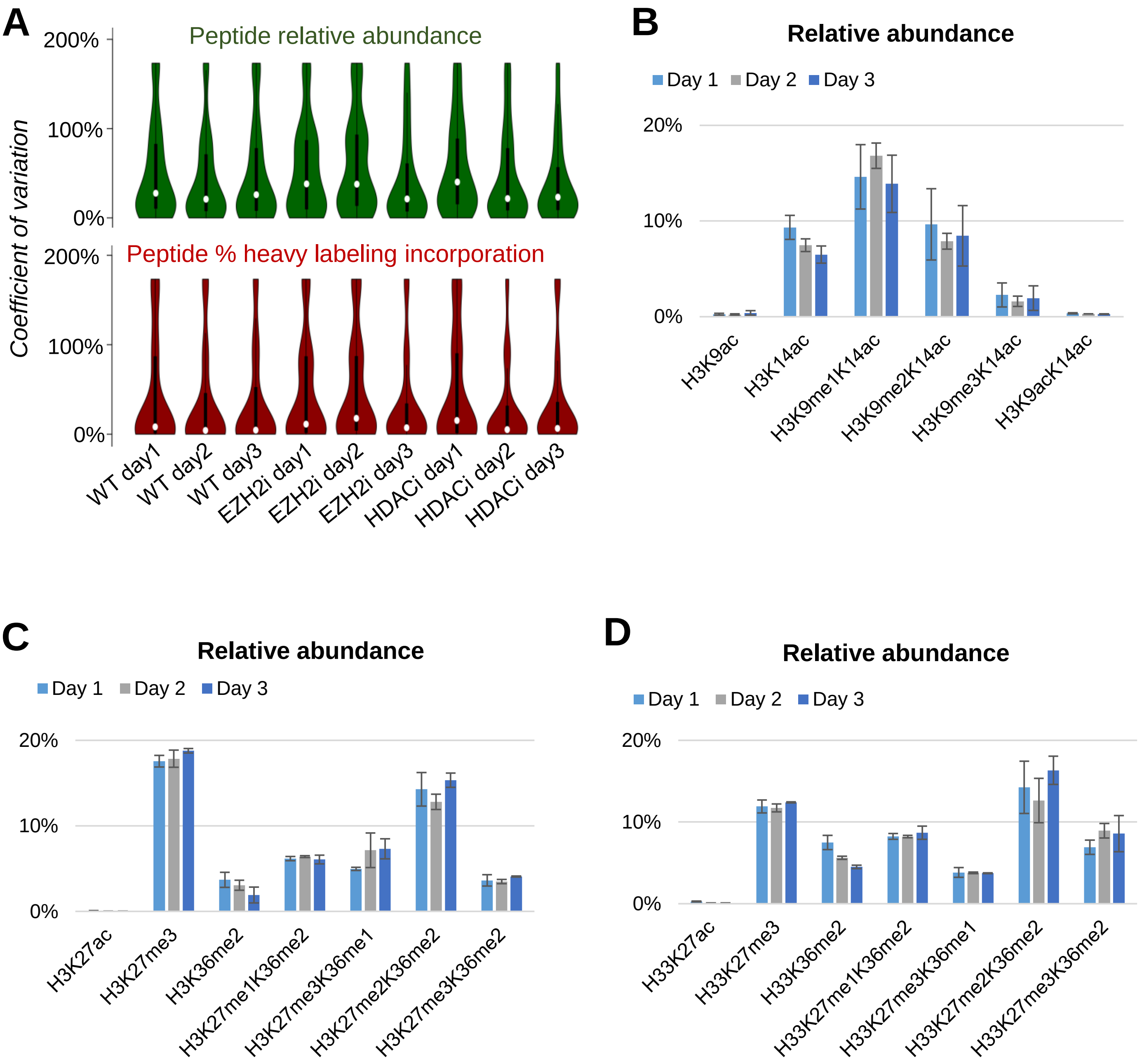

Figure S1: **Peptide data distribution and highlight of abundant co-existing PTMs on three days cell growth.** ***(A)*** Distribution of the coefficient of variation (CV) of three replicates for each of the 9 samples (three days, one wild type and two drug treatments). The green violin plot represents the CV of the calculated peptide relative abundance; the red violin plot represents the CV of the labeling incorporation. ***(B)*** Relative abundance of all the modified forms of the peptide of histone H3 aa 9-17. ***(C)*** Relative abundance of modified forms of the peptide of histone H3 aa 27-40 and ***(D)*** same peptides for the variant H3.3 (A31S).

Figure S2

Correlation	1day - abundance - 1	1day - abundance - 2	1day - abundance - 3	2day - abundance - 1	2day - abundance - 2	2day - abundance - 3	3day - abundance - 1	3day - abundance - 2	3day - abundance - 3	1day - labeling - 1	1day - labeling - 2	1day - labeling - 3	2day - labeling - 1	2day - labeling - 2	2day - labeling - 3	3day - labeling - 1	3day - labeling - 2	3day - labeling - 3
1day - abundance - 1	1.00																	
1day - abundance - 2	0.92	1.00																
1day - abundance - 3	0.91	0.98	1.00															
2day - abundance - 1	0.94	0.92	0.92	1.00														
2day - abundance - 2	0.95	0.92	0.92	0.97	1.00													
2day - abundance - 3	0.85	0.91	0.90	0.84	0.86	1.00												
3day - abundance - 1	0.95	0.93	0.93	0.98	0.98	0.84	1.00											
3day - abundance - 2	0.94	0.93	0.95	0.96	0.96	0.84	0.98	1.00										
3day - abundance - 3	0.91	0.96	0.98	0.93	0.94	0.87	0.95	0.97	1.00									
1day - labeling - 1	0.09	0.09	0.09	0.09	0.11	0.08	0.09	0.09	0.09	1.00								
1day - labeling - 2	0.15	0.14	0.12	0.11	0.13	0.11	0.12	0.12	0.12	0.59	1.00							
1day - labeling - 3	0.05	0.07	0.07	0.08	0.07	0.07	0.06	0.07	0.07	0.59	0.74	1.00						
2day - labeling - 1	0.14	0.11	0.08	0.11	0.12	0.10	0.11	0.10	0.09	0.66	0.71	0.59	1.00					
2day - labeling - 2	0.18	0.16	0.15	0.20	0.18	0.14	0.17	0.17	0.15	0.65	0.79	0.72	0.76	1.00				
2day - labeling - 3	0.14	0.15	0.12	0.16	0.15	0.16	0.14	0.13	0.13	0.50	0.76	0.72	0.58	0.80	1.00			
3day - labeling - 1	0.17	0.15	0.14	0.15	0.17	0.14	0.16	0.16	0.14	0.56	0.68	0.58	0.63	0.73	0.63	1.00		
3day - labeling - 2	0.17	0.15	0.15	0.15	0.16	0.12	0.15	0.17	0.16	0.60	0.71	0.63	0.79	0.81	0.66	0.79	1.00	
3day - labeling - 3	0.15	0.15	0.16	0.17	0.14	0.15	0.14	0.15	0.16	0.51	0.69	0.59	0.55	0.71	0.60	0.64	0.60	1.00

Figure S2: **Correlation between histone peptide relative abundance and labeling rate.** R correlation between all biological replicates and days of growth in heavy labeled media (1-3) of untreated EL4 cells. R correlation values potentially span between -1 and +1. A correlation close to 0, i.e. abundance vs labeling rate, correspond to no correlation between the two datasets.

Figure S3

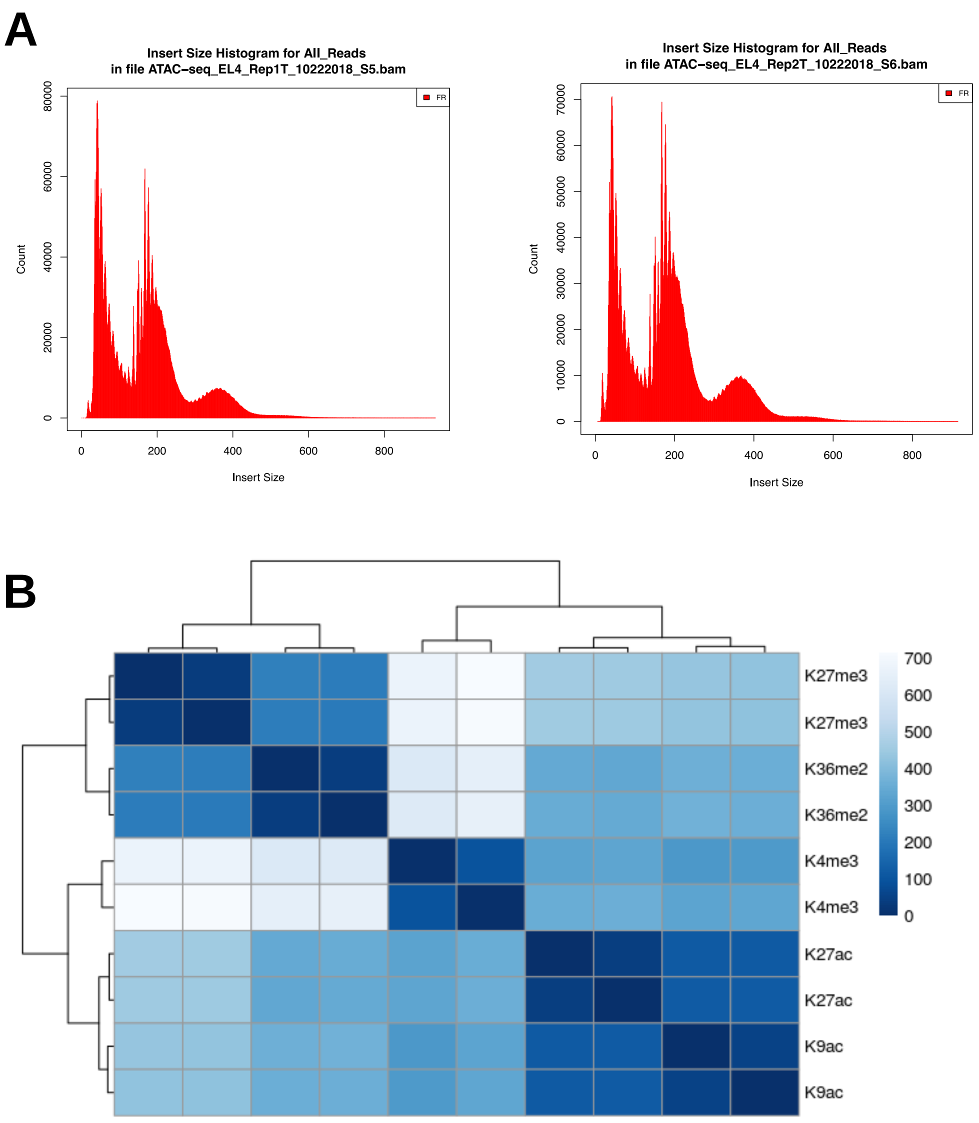

Figure S3: **Quality control of genomics analysis.** ***(A)*** Distribution of ATAC-seq fragment sizes for replicate 1 (left) and replicate 2 (right). ***(B)*** Correlation analysis of mapped genomics locations for the five selected histone H3 marks, each analyzed in two biological replicates.

Figure S4

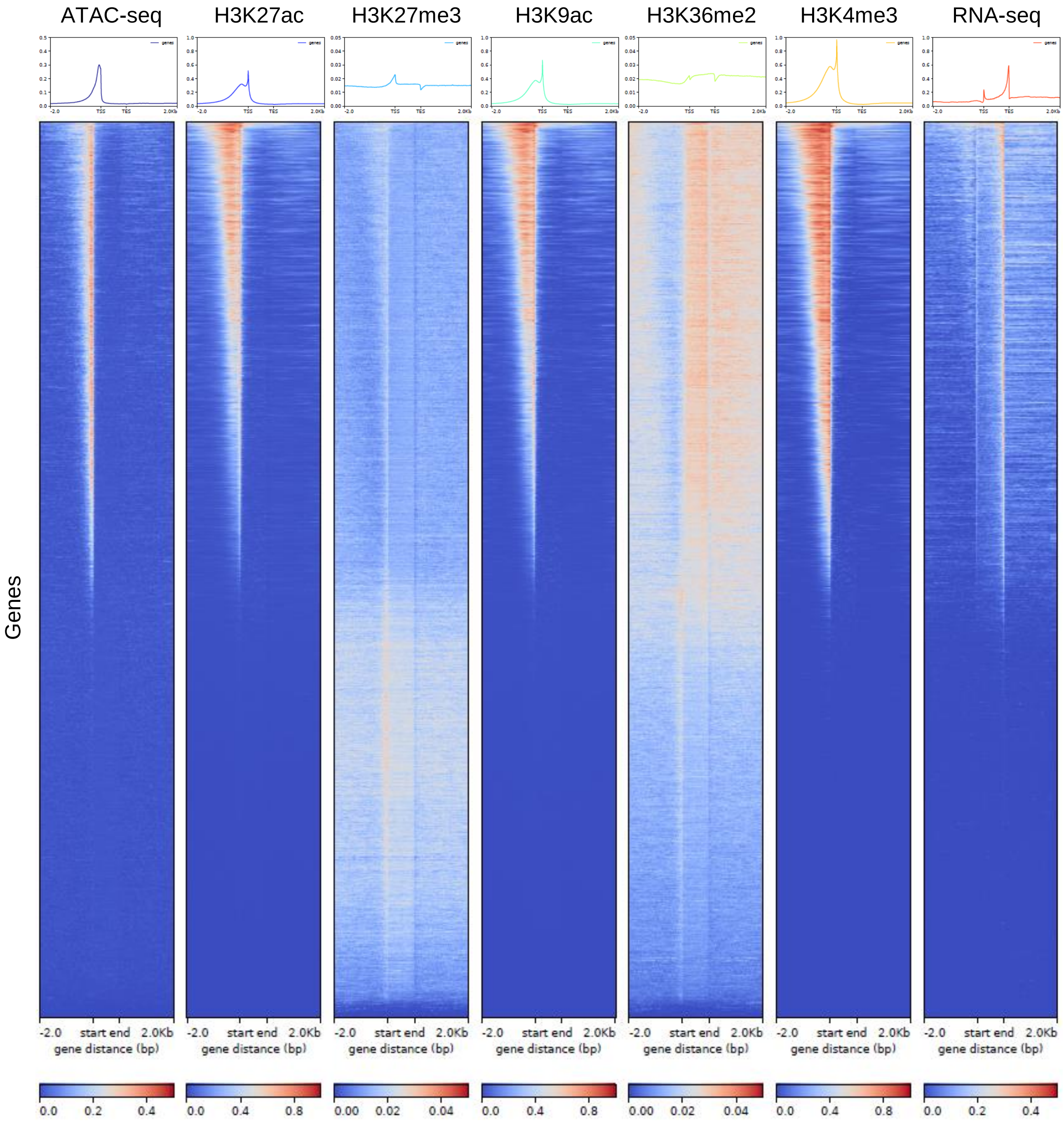

Figure S4: **Heatmaps of genomics peak intensities sorted by ATAC-seq reads. On the top, histogram of signal intensity around gene promoters (start), end of the gene (end) or nearby locations (±2 kb).**

Figure S5

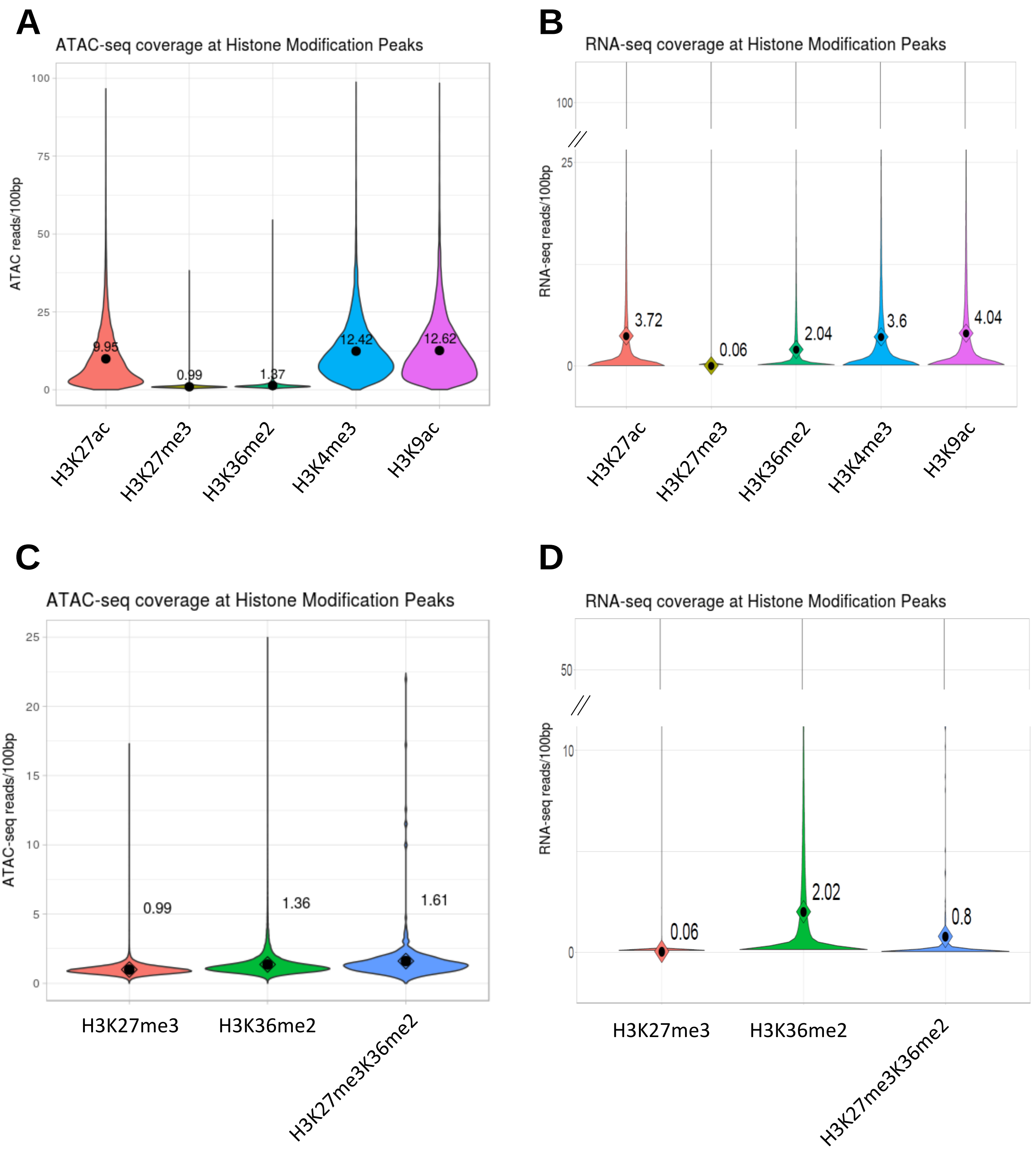

Figure S5: **Density plots of ChIP-seq peak widths intersecting with ATAC-seq or RNA-seq.** ***(A)*** ATAC-seq and ***(B)*** RNA-seq reads per 100 bp distribution of the five ChIP-seq experiments of the selected histone H3 PTMs. Results are average of two biological replicates. ***(C)*** Selected analysis of genomics loci mapped for H3K27me3 ChIP, H3K36me2 ChIP and the intersected ChIP-seq domains where both marks mapped. The intersection is performed with ATAC-seq and ***(D)*** RNA-seq reads.

Figure S6

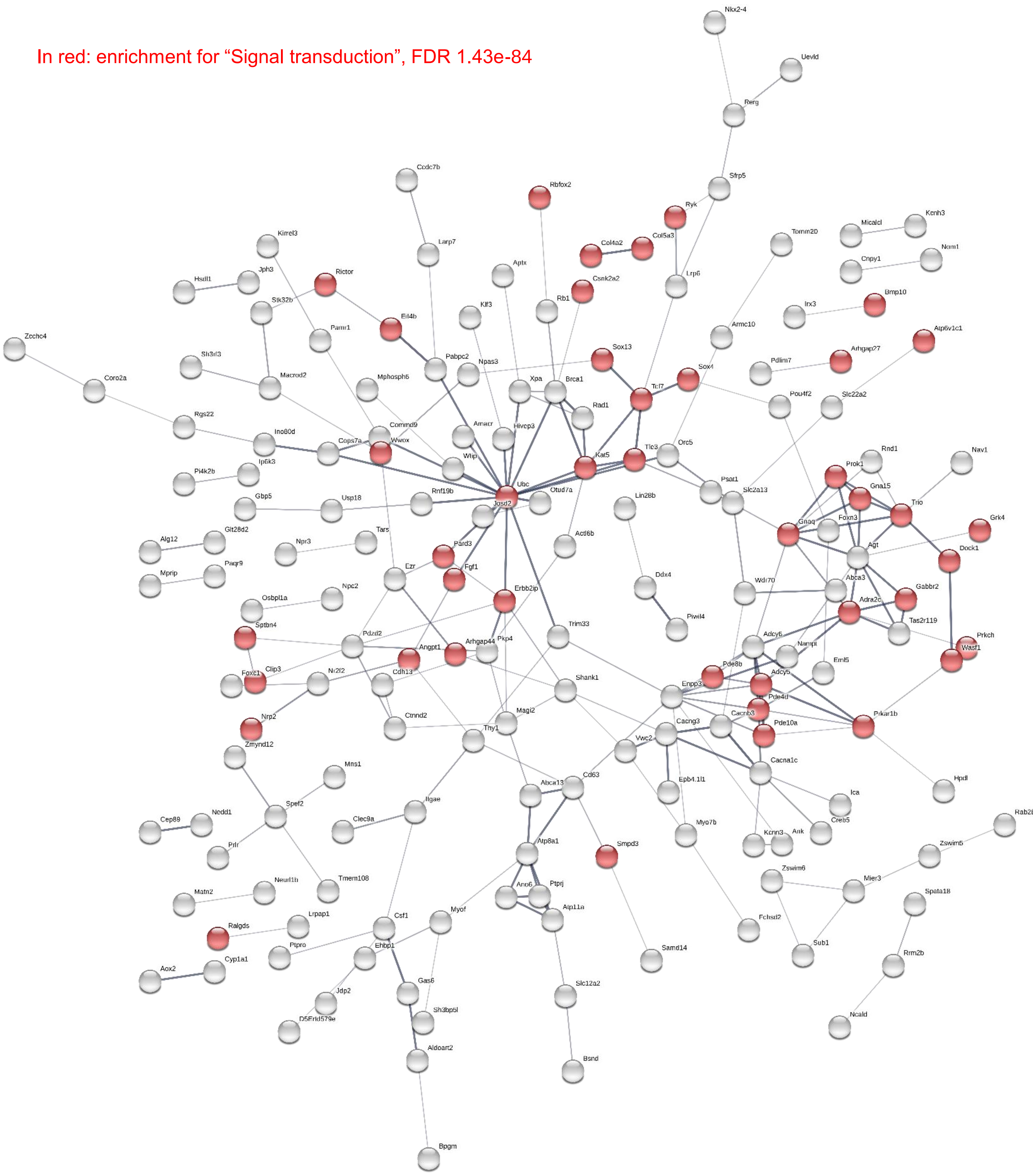

Figure S6: **STRING analysis (http://string-db.org) of genes mapping nearby the peaks obtained from the intersection of H3K27me3 and H3K36me2 ChIP-seqs.** Genes in red were those corresponding to the annotation “signal transduction” (Reactome Pathways).

Figure S7

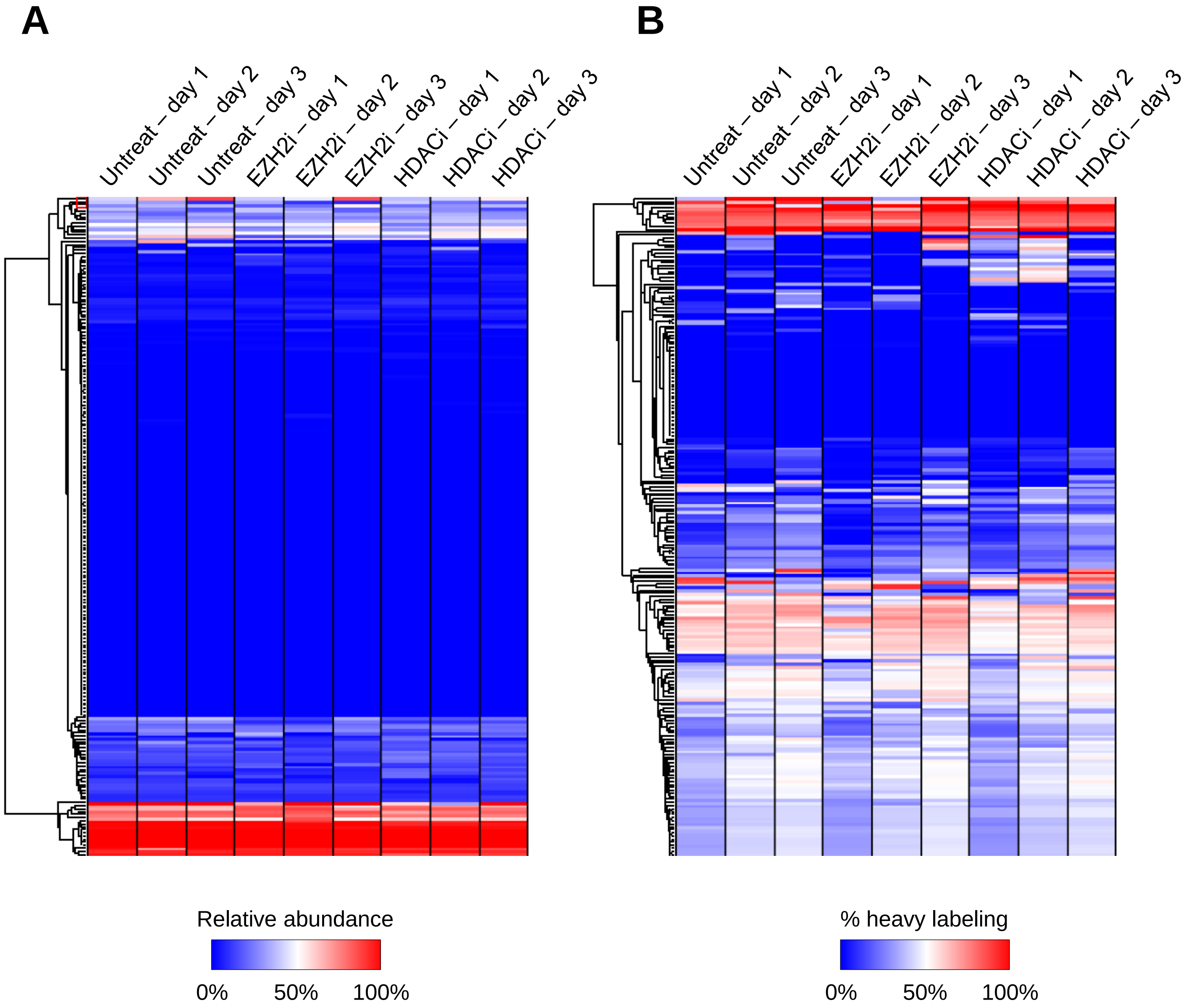

Figure S7: **Heatmap of all relative abundances and % labeling incorporation.** ***(A)*** Overview of all relative abundances of histone peptides quantified at three days of EL4 cell growth without treatment, treated with EZH2 inhibitor and HDAC inhibitor. ***(B)*** Display of the percentage of heavy labeling in the same conditions.

Figure S8

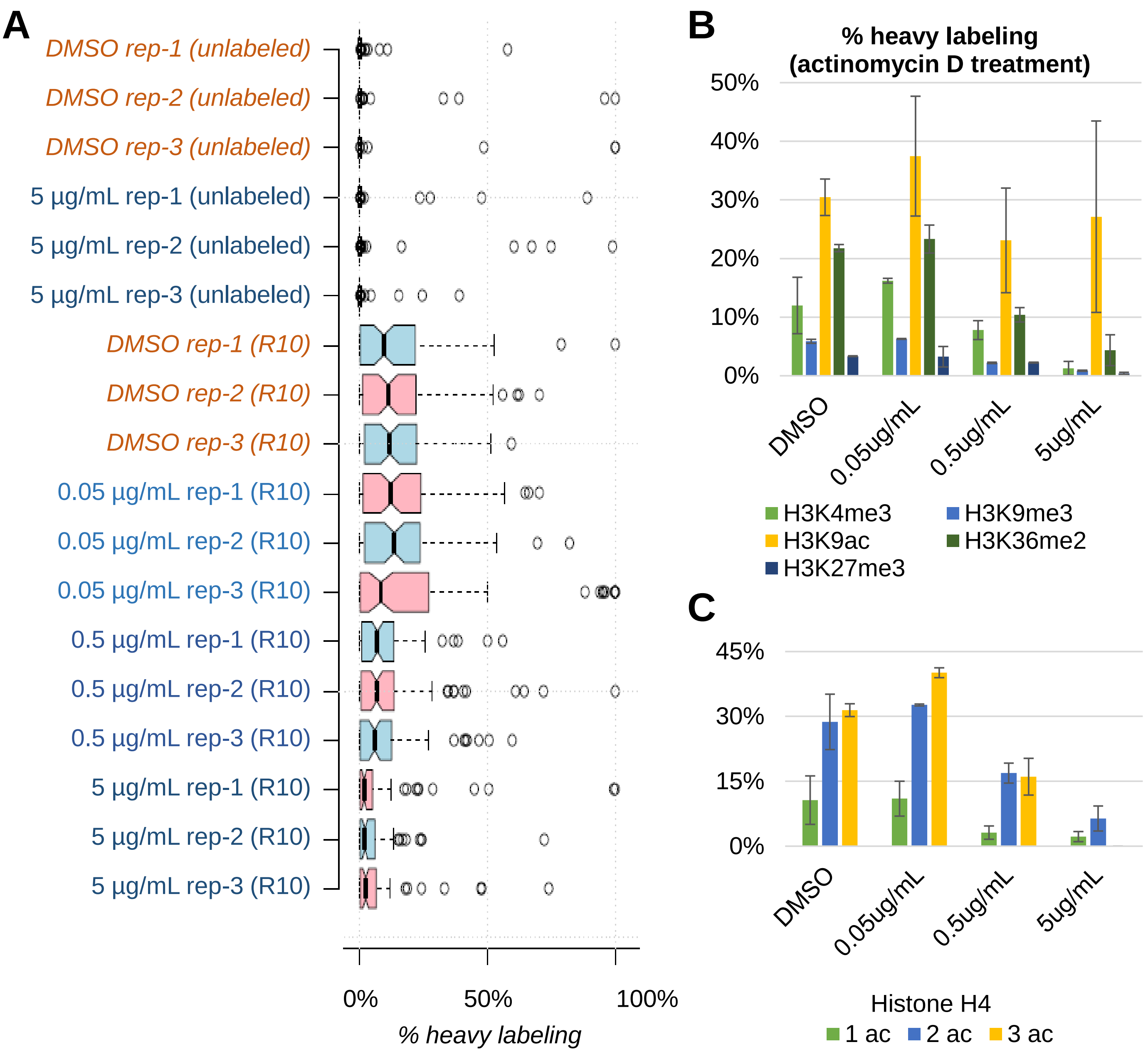

Figure S8: **Box plot representing the heavy labeling incorporation on histone H3 and H4 peptides upon actinomycin D treatment.** ***(A)*** Actinomycin is a drug responsible for transcriptional inhibition. It binds DNA at the transcription initiation complex and prevents elongation of RNA chain by RNA polymerase. We included in EL4 cell media heavy arginine (R10) in presence of actinomycin D using doses of 0.05, 0.5, and 5.0 µg/mL for roughly 17 hrs. Cells were treated in a 24 well plate with 1 mL per well and 1 million cells per well. Some wells received unlabeled arginine as a labeling control (unlabeled). Overall, we show that higher concentrations of actinomycin D corresponds to a lower heavy labeling incorporation. ***(B)*** No significant differences in the ranking of labeling incorporation was observed for the 5 investigated histone H3 peptides and ***(C)*** for the acetylation state of histone H4.

Figure S9

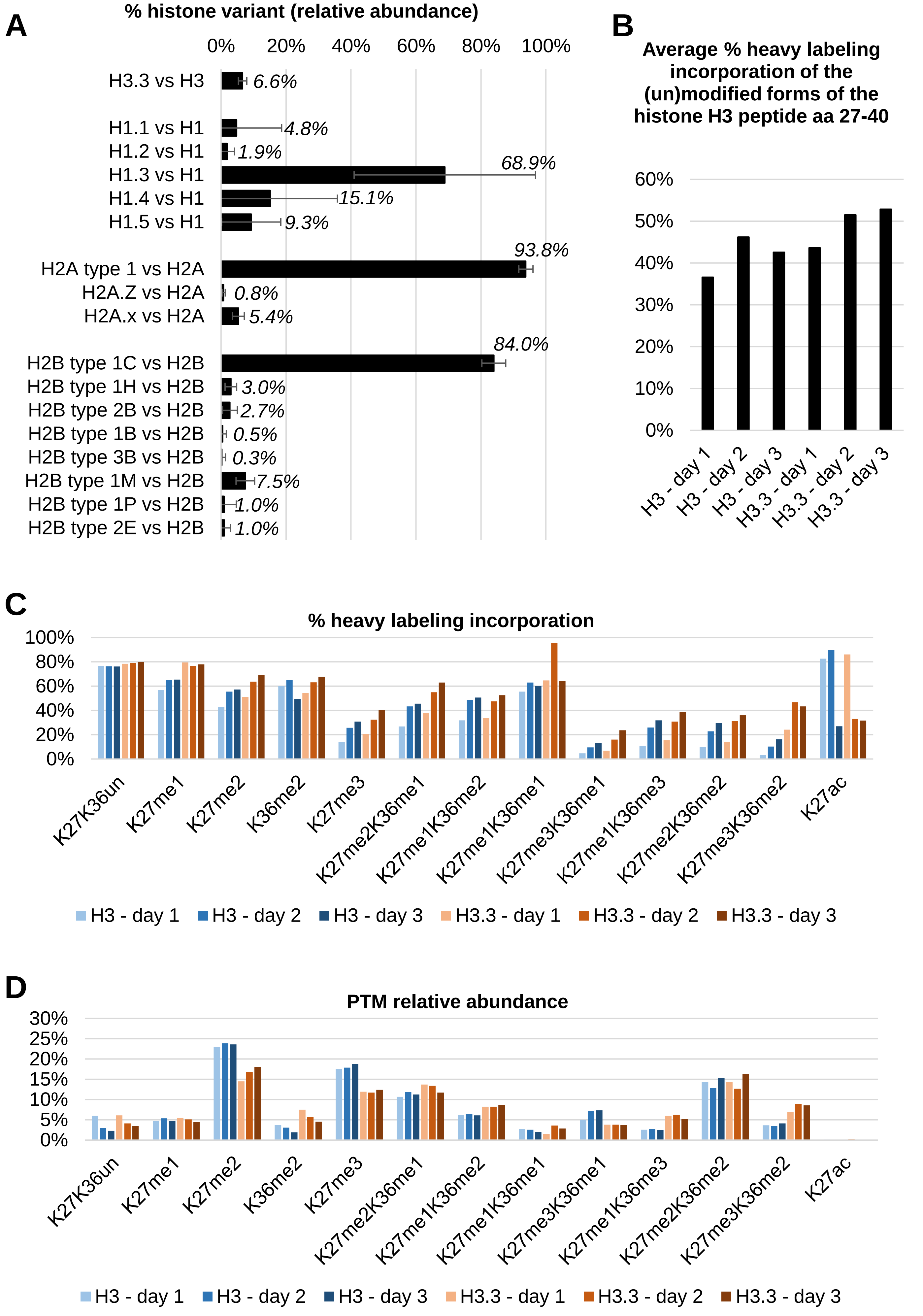
A
% histone variant (relative abundance)
0%
20%
40%
60%
80%
100%
H3.3 vs H3
6.6%
H1.1 vs H1
4.8%
H1.2 vs H1
1.9%
H1.3 vs H1
68.9%
H1.4 vs H1
15.1%
H1.5 vs H1
9.3%
H2A type 1 vs H2A
93.8%
H2A.Z vs H2A
0.8%
H2A.x vs H2A
5.4%
H2B type 1C vs H2B
84.0%
H2B type 1H vs H2B
3.0%
H2B type 2B vs H2B
2.7%
H2B type 1B vs H2B
0.5%
H2B type 3B vs H2B
0.3%
H2B type 1M vs H2B
7.5%
H2B type 1P vs H2B
1.0%
H2B type 2E vs H2B
1.0%
B
Average % heavy labeling incorporation of the (un)modified forms of the histone H3 peptide aa 27-40
0%
10%
20%
30%
40%
50%
60%
H3 - day 1
H3 - day 2
H3 - day 3
H3.3 - day 1
H3.3 - day 2
H3.3 - day 3
C
% heavy labeling incorporation
0%
20%
40%
60%
80%
100%
K27K36un
K27me1
K27me2
K36me2
K27me3
K27me2K36me1
K27me1K36me2
K27me1K36me1
K27me3K36me1
K27me1K36me3
K27me2K36me2
K27me3K36me2
K27ac
H3 - day 1
H3 - day 2
H3 - day 3
H3.3 - day 1
H3.3 - day 2
H3.3 - day 3
D
PTM relative abundance
0%
5%
10%
15%
20%
25%
30%
K27K36un
K27me1
K27me2
K36me2
K27me3
K27me2K36me1
K27me1K36me2
K27me1K36me1
K27me3K36me1
K27me1K36me3
K27me2K36me2
K27me3K36me2
K27ac
H3 - day 1
H3 - day 2
H3 - day 3
H3.3 - day 1
H3.3 - day 2
H3.3 - day 3

Figure S9

Figure S9: **Analysis of relative abundance and labeling rate of histone variants at 1-3 days of EL4 cell growth.** ***(A)*** Relative abundance of histone variants compared to the total abundance of a given histone family (e.g. H3.3 vs total H3), calculated for those histones which workflow produces peptides with unambiguous sequence. ***(B)*** Average heavy labeling of all peptides unique for histone H3.3 vs canonical H3 variants, i.e. the peptide aa 27-40, KSAPATGGVKKPHR vs KSAPSTGGVKKPHR. ***(C)*** Relative labeling incorporation for the differentially modified forms of the same peptide with the canonical H3 sequence and the sequence variant of H3.3. ***(D)*** Relative abundance of the PTMs on histone H3 vs H3.3.
